# Proximal Soil Sensing – A Contribution for Species Habitat Distribution Modelling of Earthworms in Agricultural Soils?

**DOI:** 10.1371/journal.pone.0158271

**Published:** 2016-06-29

**Authors:** Michael Schirrmann, Monika Joschko, Robin Gebbers, Eckart Kramer, Mirjam Zörner, Dietmar Barkusky, Jens Timmer

**Affiliations:** 1 Leibniz Institute for Agricultural Engineering Potsdam-Bornim, Max-Eyth-Allee 100, 14469, Potsdam, Germany; 2 Leibniz Centre for Agricultural Landscape Research (ZALF), Institute for Landscape Biogeochemistry, Eberswalder Str. 84, 15374, Muencheberg, Germany; 3 Eberswalde University for Sustainable Development, Schicklerstraße 5, 16225, Eberswalde, Germany; 4 Forschungszentrum Juelich GmbH, Institute of Bio- and Geosciences, IBG-3, Wilhelm-Johnen-Straße, 52428, Juelich, Germany; 5 Leibniz Centre for Agricultural Landscape Research (ZALF), Research Station Muencheberg, Eberswalder Str. 84, 15374, Muencheberg, Germany; 6 University of Freiburg, BIOSS Centre for Biological Signalling Studies, Schänzlestr. 18, 79104, Freiburg, Germany; DOE Pacific Northwest National Laboratory, UNITED STATES

## Abstract

**Background:**

Earthworms are important for maintaining soil ecosystem functioning and serve as indicators of soil fertility. However, detection of earthworms is time-consuming, which hinders the assessment of earthworm abundances with high sampling density over entire fields. Recent developments of mobile terrestrial sensor platforms for proximal soil sensing (PSS) provided new tools for collecting dense spatial information of soils using various sensing principles. Yet, the potential of PSS for assessing earthworm habitats is largely unexplored. This study investigates whether PSS data contribute to the spatial prediction of earthworm abundances in species distribution models of agricultural soils.

**Methodology/Principal Findings:**

Proximal soil sensing data, e.g., soil electrical conductivity (EC), pH, and near infrared absorbance (NIR), were collected in real-time in a field with two management strategies (reduced tillage / conventional tillage) and sandy to loam soils. PSS was related to observations from a long-term (11 years) earthworm observation study conducted at 42 plots. Earthworms were sampled from 0.5 x 0.5 x 0.2 m³ soil blocks and identified to species level. Sensor data were highly correlated with earthworm abundances observed in reduced tillage but less correlated with earthworm abundances observed in conventional tillage. This may indicate that management influences the sensor-earthworm relationship. Generalized additive models and state-space models showed that modelling based on data fusion from EC, pH, and NIR sensors produced better results than modelling without sensor data or data from just a single sensor. Regarding the individual earthworm species, particular sensor combinations were more appropriate than others due to the different habitat requirements of the earthworms. Earthworm species with soil-specific habitat preferences were spatially predicted with higher accuracy by PSS than more ubiquitous species.

**Conclusions/Significance:**

Our findings suggest that PSS contributes to the spatial modelling of earthworm abundances at field scale and that it will support species distribution modelling in the attempt to understand the soil-earthworm relationships in agroecosystems.

## Introduction

Understanding the spatial variation of soil biota and its abiotic and biotic drivers is a keystone for recognizing the soil ecosystem functioning and its influences on ecosystem services the soil environment provides [1,2]. Spatial patterning of soil biota ranges from the micro scale (mm) to the regional scale (km) and strongly depends on the hierarchical nested soil variation occurring in space [1,3,4]. Species distribution models (SDM) provide valuable insights into the drivers of these spatial patterns over a wide range of scales [3]. These models help to understand or predict the spatial distribution of species and their abundances by using a careful selection of predictors and sufficient observations [5].

For collecting soil data more efficiently at the field scale, soil sensors and mobile sensor platforms have been recently developed. They allow for spatially dense mapping of physico-chemical soil properties on cultivated land and may help to improve SDM and their output. Field scale is defined as the geographical dimension of an agricultural field, typically ranging from 1 ha to 100 ha. According to the theory of geographical dimension, which is rooted in landscape ecology [6], the scale of spatial studies (encompassing extent, support, coverage) is linked to the methods and models used for describing the spatial processes [7]. The field scale requires observation within distances of several meters. The optimum sampling intervals for assessing spatial patterns can be derived by geostatistical analysis [8,7]. For covering large fields it can become too costly and time consuming to use standard methods for sampling and analysis. In particular, the investigation of soil properties, including soil biota, can be extremely laborious. Thus, sensors able to collect spatially dense data in situ and in real-time may aid ecological studies at the field scale. The possibility of dense sampling can even outweigh the limitations of the sensor approach concerning precision or bias [9]. If sensors are employed close to the soil’s surface or within the soil, the approach is referred to as proximal soil sensing (PSS). A wide range of sensors including diffuse reflectance spectrometers, electrochemical sensors, electromagnetic induction sensors, ground penetrating radars, and gamma ray spectrometers is used to collect soil data in high spatial resolution, e.g., soil organic matter, pH, or texture [10]. In particular, geo-electrical sensors have a long history of soil mapping in agriculture [11]. Since soil data can be collected in real-time while driving through the field, a high density of measurement points can be obtained that suitably captures the spatial heterogeneity of soil properties [12]. Thus, PSS might provide more comprehensive soil maps compared to those from conventional soil sampling, which may give new insight into soil variation and soil processes that occur at the field scale. So far, PSS has been used to investigate abiotic soil properties such as water content, texture, pH, organic matter, and plant nutrients [13,14,15]. To the best of our knowledge, soil biological characteristics have rarely been studied by PSS.

Earthworms are ideal candidates for investigation by PSS because they are deeply connected with the soil environment. There benefits to soils and plants were recognized as early as the 19^th^ century by Charles Darwin [16]. Through their digesting, casting, and burrowing activity earthworms structure the soil, augment drainage, improve aggregate stability, mix organic material with mineral soil, contribute to the formation of humic substances, and raise the plant availability of nutrients [17,18]. They play a major role in the soil carbon and nitrogen turnover and increase soil fertility. Earthworms are therefore well appreciated as soil or ecosystem engineers for their contribution to ecosystem services [2,17]. As many earthworms burrow in and consume soil, their abundance and species composition are strongly affected by abiotic soil properties [18]. Specifically, anecic and endogeic species are tightly bound to the soil environment. Anecic species are mostly large earthworms that produce and live in persistent vertical burrows and feed on litter on the surface. Endogeic earthworms live in horizontal burrows and feed directly on the soil. Effects on earthworms by soil texture, pH, temperature, and compaction as well as the content of organic matter, water, and heavy metals have been observed [19]. With their diet consisting of plant and root residues in various stages of decay, they prefer soils enriched with organic materials [20]. Most species favor soils with pH values ranging from slightly acidic to slightly alkaline and are rarely found in soils with pH < 4 [21]. Anthropogenic activities such as tillage in arable soils influence earthworm activity as well [22,23].

Spatial analyses using semivariograms indicated that a high spatial heterogeneity of earthworm abundances exists on field scale [23,24,25,26,27,28]. The semivariogram is an established tool in spatial ecology to describe the behavior of dissimilarity between sampling points of species abundances depending on their spatial distances [7]. Nuutinen et al. (2011) [25] stated that even by using a 25 x 25 m² sampling grid much of the small scale variation of earthworm abundances could not be captured. Since accurate observation of earthworm activity involves time-consuming hand sorting, spatial investigations of earthworm distributions are often restricted to a limited number of samples and thus do not sufficiently cover the study area.

If relationships between earthworm habitat factors and soil sensor measurements could be established, PSS might become a useful approach for investigating the spatial distribution of earthworm abundances in arable soil. So far, only geo-electrical sensors for measuring soil apparent electrical conductivity (ECa) were investigated to address earthworm abundances. Soil ECa is a general measurement of conductance through a volume of soil and it is therefore related to many soil properties that provide current pathways, e.g., soil salinity, saturation, water content, bulk density, clay content, and organic matter [7]. Under non-saline anhydromorphic conditions, soil ECa has been used to differentiate soil texture within fields [29,30]. Valckx et al. (2009) [27] observed relations between soil ECa and the abundance of deep burrowing anecic earthworm species (*Aporrectodea longa* and *Lumbricus terrestris*). Studies also showed the relationships between soil ECa and earthworm abundances that were additionally influenced by the soil management system [31,32]. While Valckx et al. (2009) [27] and Lardo et al. (2012) [32] used electromagnetic induction (EMI) to obtain ECa readings, Joschko et al. (2010) [31] applied the galvanic coupled electrical resistivity (GCR) method [30]. The EMI method is based on the soil conductivity dependent modification of the electromagnetic field emitted by the instrument. These EMI instruments do not require to be in direct contact with the soil and they are usually relatively small (1 m length) and light weighted (about 7 kg). The GCR instruments use at least four electrodes, which have to be in direct (galvanic) contact with the soils for measuring electrical resistivity of the ground by the so called four-point method [30]. If GCR instruments are mounted on a platform, which can be pulled by a vehicle, these systems are larger und much heavier (several 100 kg) than EMI systems. If the electrode spacing is selected adequately, both of the geo-electrical methods should provide the same information (electrical conductivity is the inverse of electrical resistivity). However, EMI systems are more sensitive to ambient electromagnetic disturbances while GCR are facing problems when the soil is very dry and contact resistance becomes high.

The previously mentioned studies confirmed that soil ECa aided in determining the driving factors of the earthworm distribution. Apart from geo-electrical sensing, optical and electrochemical soil sensors may help to contribute to the knowledge of earthworm spatial distribution. They can address relevant factors affecting earthworms such as soil pH, organic carbon, and nutrient content [33,34]. In this regard, the role of PSS has still to be studied more precisely. Within SDM, PSS could help to improve the prediction of earthworm abundances on cultivated land. Improved maps will then add to the linking between earthworm observations and environmental estimates to highlight causal ecological drivers that govern the earthworm activity. Those maps will eventually provide guidance for soil biological studies and reduce sampling effort.

The objective of this study was to examine whether PSS may contribute as a spatial predictor for the modelling of earthworm abundances in SDM of agricultural soils. Previous research clarified the sensor–soil relationships and the earthworm–soil relationships. Therefore, we expected that a quantitative relationship between earthworm abundances and PSS data exists. We aimed to explore the correlations of a wide range of proximal soil sensors, e.g., a geo-electrical sensor, a pH-meter, and a spectrometer, with the potential of earthworm abundances in a field with sandy to loam soils. We used then generalized additive models and state-space modelling to predict earthworm abundances at the field scale. As prediction we generally refer to spatial prediction throughout this paper. The findings suggest that PSS will support the modelling of the spatial distribution of earthworms and that it would become a valuable spatial predictor in species distribution models on cultivated land for analysing and predicting earthworms.

## Materials and Methods

### Study area and earthworm sampling

**Fig 1:**
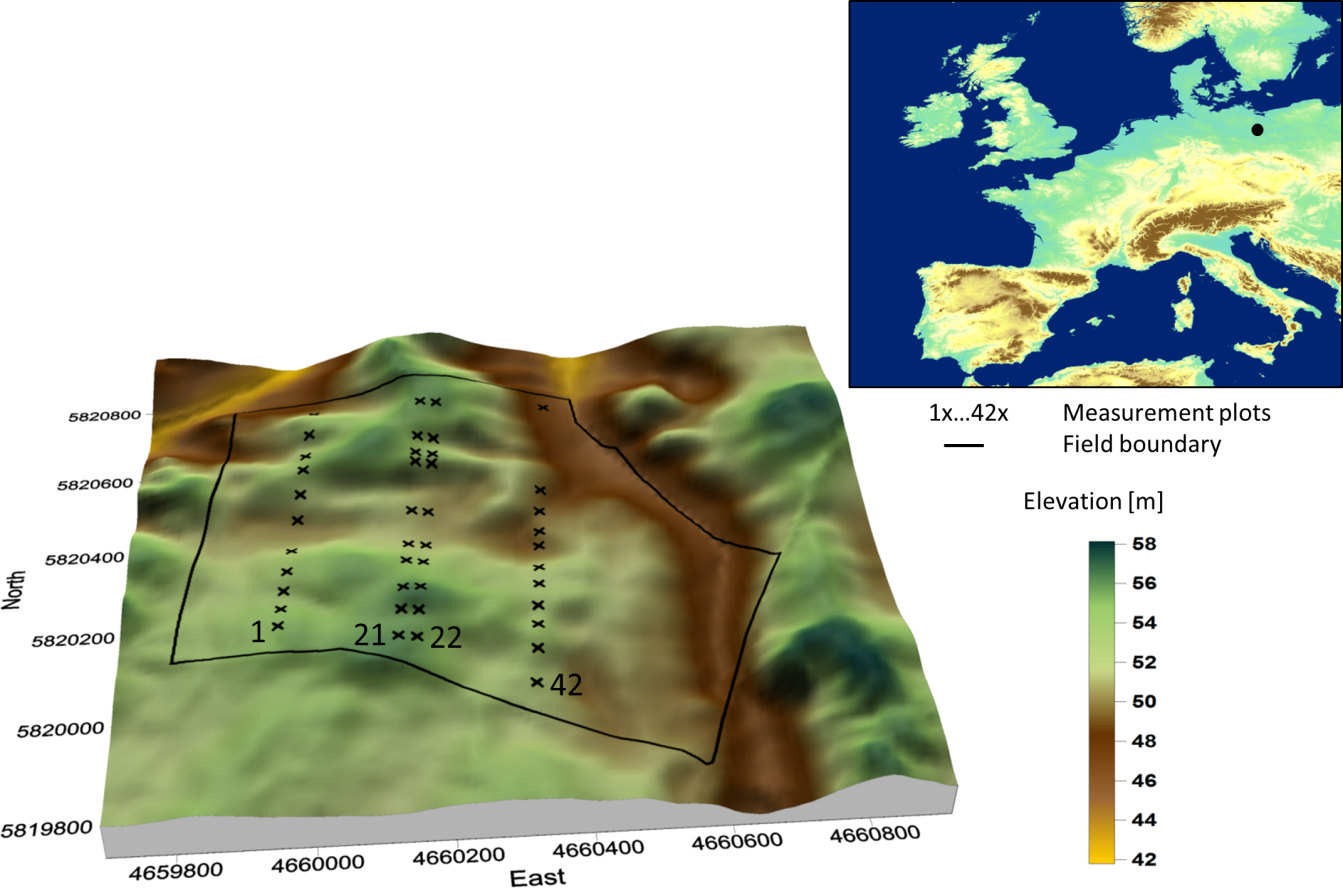
Earthworm measurement plots. The undulating terrain of the field near Lietzen is typical for the landscape formed by the last Ice-age formed landscape in Northeastern Germany. The 42 measurement plots were distributed along four transects in the 63 ha field with 1–21 being under conventional and 22–42 under reduced tillage. The average distance between plots within the transects was 66 m.

The study was conducted within a 63 ha field in the state of Brandenburg, Germany (52° 29’ N, 14° 21’ E) with kind permission of Gebhard Graf von Hardenberg (Komturei Lietzen GmbH) [23,31]. The field is governed by the glacial and periglacial forming processes of the Weichsel glaciation. Within the smoothly rolling ground moraine landscape sandy topsoil typically covers loamy subsoil and the soil heterogeneity is strongly interrelated with terrain and geology. The soils are classified as luvisols and the topsoil layer consists of a variable mix of sand and loam. Elevation varies between 45 and 57 m. Elevation declines towards the eastern border where a small creek drains the field (Fig 1). The climate is characterized by 9.6°C mean annual temperature and 472 mm mean annual rainfall. The crop rotation of the field is cereal-dominated.

The field had been under conventional tillage until 1996 before soil management strategy was changed. From September 1996 until now, non-inverting, ploughless tillage has been conducted in the eastern part of the field with a tillage depth between 0.15 and 0.18 m (reduced tillage), whereas the western part was plowed at a depth of 0.30 m (Fig 1). Amounts of fertilizer and pesticides were the same in both tillage systems except in 1997 and 1998, when additional herbicides were applied in the reduced tillage system [23]. Information about crop-rotation and carbon stock development at the Lietzen site can be found in Joschko et al. (2012) [31].

Long-term earthworm monitoring was conducted between the years 1997 and 2007. Within the field, 42 monitoring plots (2 x 15 m) were permanently installed along four transects following the main slope and tillage direction with 21 plots for each tillage type [35]. In order to minimize edge effects, the plots were positioned at least 45 m from the field borders. At each monitoring plot, a 0.5 x 0.5 x 0.2 m^3^ soil block was retrieved from the topsoil. From those blocks earthworms were hand sorted and identified to species level [36]. Earthworms were sampled in spring, and partially in autumn, each year at 42 plots between 1997 and 2007. It is known that earthworm activity is affected by soil moisture. Therefore, earthworm sampling was aimed to conduct under optimal soil conditions for earthworm activity approx. at 10% soil moisture.

### Sensor measurements

The measurements were conducted during dry and warm weather conditions from 15^th^ to 22^nd^ of August 2012. The entire field was mapped by using a combination of three different proximal soil sensors (Veris Technologies, Salinas, KS, USA). The assembly included a geo-electrical sensor, an electrochemical sensor, and an optical sensor for measuring apparent electrical conductivity (ECa), pH, and diffuse reflectance in the near infrared (NIR). They were mounted on a single platform which was attached to a tractor. The geo-electrical sensor consisted of six coulter electrodes arranged in a Wenner array configuration and recorded soil ECa at a shallow depth (ECa_sh_) and a deeper depth (ECa_dp_), e.g., 70% of the signal were coming from 0.12 and 0.37 m, respectively. The pH sensor used a hydraulic system to grab soil material from 0.10 m depth and bring it into direct contact with two antimony pH electrodes. The spectrometer consisted of a fibre optic sensor for measuring soil reflection within the near infrared (NIR) spectral range (1100–2200 nm) at a resolution of about 7 nm. The sensor head was located in a shank, which was pulled through the soil at approx. 0.05 m soil depth. The bottom of the shank had a window (18 mm in diameter, covered by a sapphire glass), which was used to illuminate the soil and collect the reflected light. The light was led to the spectrometer via glass fibres. These sensors and their platform have been previously tested from our research group for mapping chemical soil properties [15,33].

Sensor measurements were carried out carefully with a speed of 3 km h^-1^. Measurement direction was aligned in parallel to the usual tramline direction. The spacing between the tramlines varied between 16 and 20 m. Soil reflectance and soil ECa was recorded every second, i.e., approx. each 1 m, whereas soil pH measurements were recorded according to the equilibrium state of the two pH electrodes, i.e., every 12 m on average. Tests of the system for pH mapping under field conditions in Germany resulted in pH maps with underlying measurement densities between 25 and 90 samples ha^-1^ and a mean absolute error between 0.28 and 0.45 [15]. Measurements had to be conducted over several days because the spectrophotometer had to be cooled down due to high air temperatures. This necessitated recalibration of the pH system each day. Recalibration was validated by 50 soil samples. Recalibration samples were analysed for soil pH (lab pH) in a CaCl_2_-soil suspension after extraction for 2 h according to German standards [37].

### Pre-processing of the sensor data

All sensor measurements, i.e., ECa_sh_, ECa_dp_, pH, and NIR spectra were cleaned from obvious outliers assisted by a geographic information system (GIS). The different runs of the pH measurements were first matched against each other and afterwards calibrated with the lab pH values. Matching was done by extrapolating measurements from two different runs onto each other with ordinary Kriging [38]. Extrapolated values along the midline of both runs were regressed by Deming regression and matching coefficients were computed in order to adjust both measurement runs. Deming regression implemented the residues of both the dependent and independent variable [39]. This procedure was repeated with all measurement runs. The correlation between lab pH and matched sensor pH was significant with r = 0.77 (p < 0.001).

From the NIR absorbance spectra, average absorbance values over the entire spectral range were calculated to preserve the effect of scattering (NIR_avg_). Most of the specific chemical soil information stored in NIR soil spectra occurs from photon absorptions due to vibrations or stretches at dipole molecules [40,41]. In order to make better use of the NIR data, we calculated the second derivative of the spectra. This should remove the so-called baseline of the spectra, i.e., most of the additive and multiplicative scattering effects, exaggerates absorption features, and differentiates overlapping absorption features with the drawback of increasing spectral noise [41]. The second derivative was applied to the spectra using the Savitzky-Golay filter with a window size of 21 bands after interpolating the spectra onto 3 nm equal wavelength bands [42]. In order to reduce dimensionality and extract prominent features of the differentiated NIR spectra, independent component analysis (ICA) was applied. ICA separates a mixed signal into its independent components by an unsupervised statistical approach. It has been previously used to identify unknown concentrations from NIR spectra [43]. We applied the FastICA algorithm on the differentiated NIR soil spectra to calculate 10 ICA components [44]. Two of these components were selected as predictor variables (NIR_ICA1_ and NIR_ICA2_), which were not prone to instrument noise as assessed by visual inspection of the respective ICA score maps.

The ECa_sh,_ ECa_dp_, pH, NIR_avg,_ NIR_ICA1_, and NIR_ICA2_ values were interpolated onto the locations of the measurement plots with a 1x1 m support size by ordinary block Kriging [38]. All semivariogram modelling was done by weighted least squares regression using either the spherical, penta-spherical or exponential semivariogram models (R package gstat, [45]). The interpolated PSS values at the measurement plots were later used as variables in the modelling process.

### Statistical analysis and modelling

We estimated the potential of earthworm abundance at each plot and for each species using the averages and total counts of the observed earthworms during the time-period 1997 and 2007. As a first assessment of the interrelationships between earthworm counts and sensor measurements, Spearman rank correlations were calculated. In a next step, we investigated the prospects of improving SDMs for earthworms with PSS by constructing global generalized additive models (GAM) between the earthworm species as response and the PSS variables as predictors. Finally, we applied local modelling of the earthworm abundances using state-space modelling in accordance to the study of Joschko et al. 2010 [31]. All data analysis was conducted with R—A Language and Environment for Statistical Computing [46].

#### Generalized additive models

GAMs are extensions to general linear models, in which the predictors can be shaped to the response via a non-parametric function [47]:

$$g(\mu )=\alpha +\sum_{j=1}^{p}f_{j}(X_{j})+\varepsilon ,$$

where g(μ) denotes the link function which describes the way the estimates μ_i_ are transformed to the predictor variables, α, ε are the intercept and error and f_j_(X_j_) are functions on the predictor variables. The GAM was set as follows:

$$ln({ew}_{i})=\alpha +f_{i}(x_{i},y_{i})+{PSS}_{i}\beta ,$$

where ew_i_ denotes the abundances of the different earthworm species observed at the measurement plots as response. PSS_i_ describes the set of sensor variables interpolated onto the measurement plots as predictors along with β representing their individual effect on the response. For handling the spatial autocorrelation among the measurement plots, a bivariate function of the spatial coordinates f_i_(x_i_,y_i_) was smoothed using thin plate regression splines [48]. The GAM was implemented with a log-link function. Prior testing of Poisson distributed models indicated overdispersion, which would eventually overestimate the significance of the effects on the response. Therefore, quasi-Poisson distributed errors were used. The best models were chosen by backward selection. Variables with p > 0.1 were deleted from the predictor set. Due to cross correlation between the variables ECa_sh_, ECa_dp_, and NIR_avg_ (we calculated generalized variance inflation factors (VIF) for those GAMs greater than 5), the GAM’s were computed separately interchanging those variables to not disregard these variables from the GAM study. Overall model quality was assessed by the R-squared (R^2^) and the generalized cross validation (GCV) score [49,50]. The R^2^ was used to quantify the explained variation throughout this study. GAM’s were calculated using the R package mgcv [50].

#### State-space analysis

Local relationships were explored by state-space analysis [51]. In state-space analysis, the variation of measurements along a sequence is assumed to be governed by an underlying state process [52].

Specifically, the state Z_i_ is related to the previous state Z_i-1_ by the state equation Z_i_ = ΦZ _i-1_ + ω_i_, where Φ is a matrix of transition coefficients and ω_i_ is an error term [52]. The state variables itself are linked to the observations Y_i_ (measurements) via the observation equation Y_i_ = M_i_Z_i_ +ω_i_, where M_i_ is a matrix of observation coefficients [52].

Since state-space analysis relies on sequential data, the sets of observations had to be arranged in a sequence which regards their spatial neighbourhood (Fig 1). Earthworm counts were square-root transformed and normalized before modelling. For state-space analysis the software STATE [53] was used.

#### Model validation

Prediction accuracy of the GAM model was validated by using leave-one-out cross validation (LOOCV). This procedure holds out one observation from the data set while the rest of the data is used for model fitting and prediction of the hold-out observation. Repeating the procedure for all observations, allows one to calculate the cross validation error (CV). Model fitting based on LOOCV provides almost unbiased results [54].

In order to compare this study to the results described in Joschko et al. (2010) [31], state-space models were evaluated with split data. One half of the data was used for model fitting whereas the other half was used for prediction (each adjacent neighboring measurement plot). For both validations, mean absolute error (MAE), mean error (ME), and R^2^ between model predictions and measurements were reported. For the individual earthworm species models, R^2^ was calculated on square-root transformed abundance data. Confidence intervals of the local models were calculated with ±2 times standard error of the local model estimates.

## Results and Discussion

### Earthworm abundances

**Fig 2:**
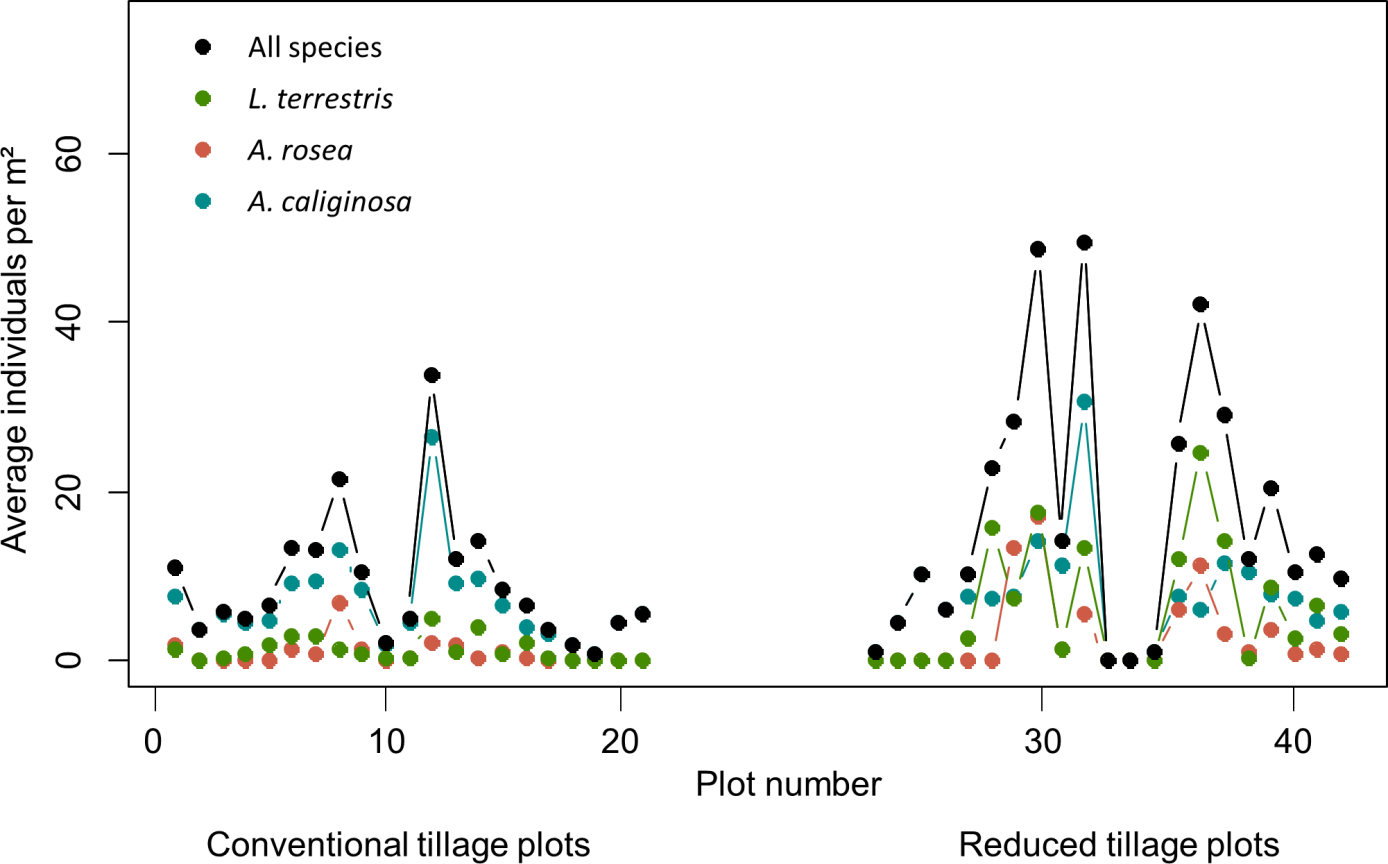
Earthworm abundances at the measurement plots. Average abundance of earthworms and of individuals for each species as observed between 1997 and 2007 at the measurement plots under conventional and reduced tillage. For configuration of plot sequence see Fig 1.

Three earthworm species were found during the long term earthworm experiment: the anecic species *L*. *terrestris* (Linnaeus 1758) and the two endogeic species *Aporrectodea caliginosa* (Savigny 1826) and *Aporrectodea rosea* (Savigny 1826) [23]. These three earthworm species are typical for tilled sandy soils in this region [4]. The spatial pattern of average earthworm abundances counted at the 42 measurement plots between 1997 and 2007 is shown in Fig 2. *Aporrectodea caliginosa* was the most abundant species, present at 40 out of 42 plots, followed by *L*. *terrestris* present at 30 plots and *A*. *rosea* was found at only 23 plots in course of the sampling period.

The average abundance of earthworms along the transects fluctuated between 0 and 49 average counts per m^2^ (Table 1). This low earthworm density corresponds to the site conditions, characterized by the low clay and carbon content and the corresponding reduced water holding capacity of the soil [23,55]. Spatial variability of earthworm abundance was higher under reduced tillage compared to conventional tillage.

**Table 1:** Univariate statistics of average individual counts of earthworm species observed at all measurement plots during the years 1997 till 2007.

	Min	Mean	Median	Max	Total sum	IQR^$^	No of zero counts
**All species**	0	13.05	10.18	49.45	6028	9.64	2
***L*.*terrestris***	0	3.73	1.27	24.72	928	3.82	12
***A*.*rosea***	0	2.01	0.36	17.09	1724	1.73	19
***A*.*caliginosa***	0	7.31	6.63	30.55	3376	4.73	2

^$^Inter quartile range

### Proximal soil sensing

**Fig 3:**
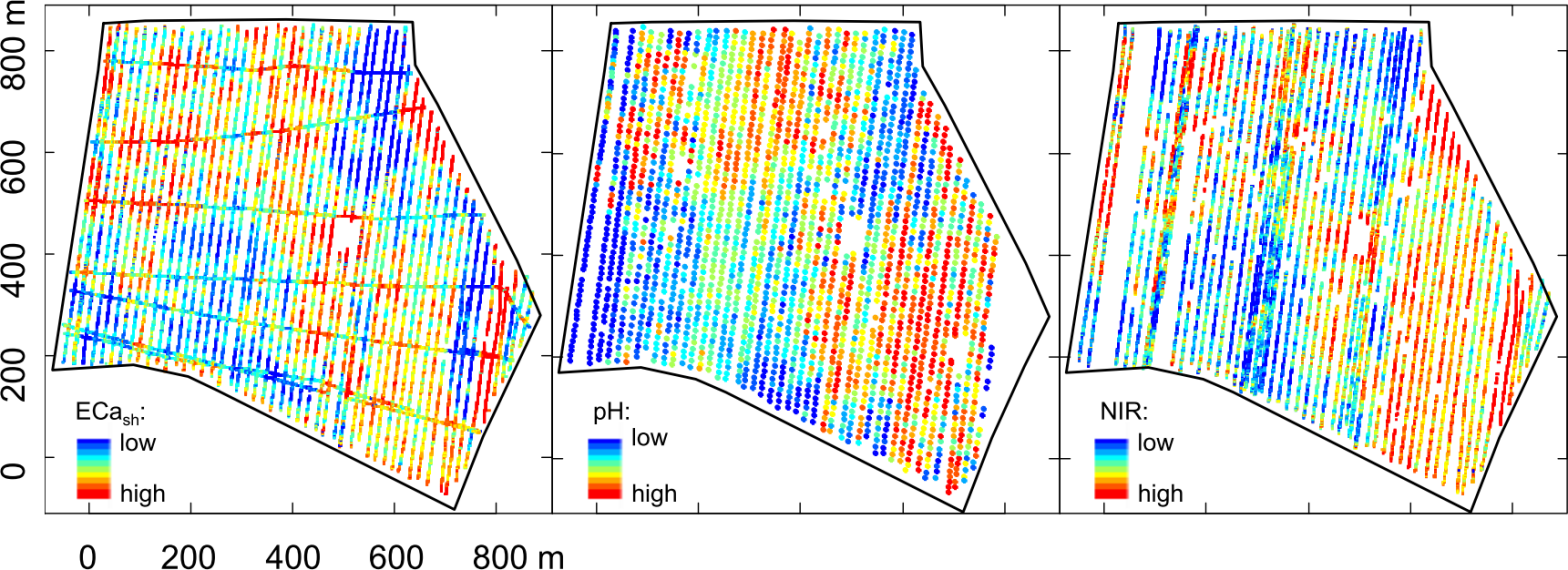
Proximal sensor measurements. Spatial locations of the sensor readings by the three-sensor platform. Apparent electrical conductivity (ECa_sh_), pH, and light absorbance (NIR_avg_) in the topsoil are depicted from left to right. Quantile classification scheme with 10 levels used.

Summary statistics and autocorrelation parameters of the sensor variables are listed in Table 2 and corresponding maps of ECa_sh_, NIR_avg_, and pH are shown in Fig 3. After removing measurement errors during post processing, the spatial density of measurement points was larger than 500 measurement points per ha for ECa and NIR and 40 measurement points per ha for pH on average. The void area in the middle to east part of the field was not accessible due to water ponding in a small depression. Missing values from the pH sensor in the utmost eastern part of the field occurred due to a system failure of the sensor electronics.

**Table 2:** Univariate statistics derived from the soil sensor data collected at the Lietzen site in summer 2011. Statistics for the variables marked with asterisk (*) were computed for the sensor values at the measurement plots interpolated with ordinary Kriging. Spectroscopic data were summarized by the average of the raw spectra (NIR_avg_) and two independent components extracted by ICA from the second derivatives (NIR_ICA1-2_). Autocorrelation range (Range) and nugget-to-sill ratio (N/S) were computed by semivariogram modelling.

Sensor	Min.	1st Qu.^$^	Median	Mean	3rd Qu.^§^	Max	Range	N/S
ECa_15_	0.20	1.20	1.60	1.84	2.10	33.40	80.39	0.00
ECa_15_*	0.61	1.21	1.54	1.81	2.13	4.06		
ECa_40_	0.30	3.20	4.50	5.38	6.50	192.10	62.95	0.01
ECa_40_*	1.34	3.52	4.54	5.19	6.73	10.82		
pH	6.24	7.27	7.34	7.34	7.43	8.02	266.70	0.00
pH*	7.19	7.30	7.34	7.35	7.42	7.48		
NIR_avg_	0.42	0.70	0.76	0.77	0.83	1.16	94.97	0.17
NIR_avg_*	0.63	0.69	0.72	0.73	0.76	1.01		
NIR_ICA1_	-2.34	-0.51	0.00	-0.10	0.35	8.32	164.15	0.02
NIR_ICA1_*	-1.02	-0.49	-0.09	-0.03	0.19	1.21		
NIR_ICA2_	-4.23	-0.52	0.00	-0.06	0.39	12.85	104.25	0.00
NIR_ICA2_*	-2.79	-0.36	0.11	0.18	0.41	3.42		

*interpolated sensor values onto measurement plots

^$^first quartile

^§^third quartile

Due to the high sand content of the soil and the dry soil conditions during the measurements, the absolute ECa_sh_ and ECa_dp_ values were low. The ECa_dp_ measurements were slightly higher and exhibited greater variability, i. e., ECa_dp_ readings showed a shorter autocorrelation range along with a small nugget effect. The map of the ECa_sh_ sensor measurements shown in Fig 3 has a distinct spatial patterning, which can be mainly explained by soil texture. In the northern part, interchanging low and high ECa_sh_ values occurred due to the relief: at the hill tops, soil material was eroded and the subsoil was exposed at the surface with higher clay content while less consolidated sandy particles accumulated downslope. This induced higher ECa_sh_ readings upslope and lower ECa_sh_ readings downslope. In the south-eastern part, ECa_sh_ values were generally lower caused by a sandy plateau located here. Strikingly high values occurred around the depression area due to higher clay content and higher soil moisture. The linear pattern of high ECa_sh_ values near the eastern field border was generated by the moisture from a small creek.

The pH distribution of the field was influenced by management and differed from the ECa pattern. In 2005 and 2007, the entire field was fertilized with lake sediments as organic amendment [35]. This treatment is known to increase the pH level in sandy soils. Therefore, pH values were high with a mean pH value of 7.34 and the variability was low with an interquartile pH range of 0.16. Despite the low variability, a distinct spatial pH pattern is visible (Fig 3). The north-western part and south-eastern part of the field were characterized by large patches of high pH values, whereas patches of low pH values were measured in the south-western part of the field. The low spatial pH variability was also confirmed by the large autocorrelation range calculated with the variogram (Table 2).

The NIR_avg_ readings showed a trend from lower absorbance in the west to higher absorbance in the east, which coincides with the catchment area of the creek at the eastern border of the field. Modelling the spatial structure of the NIR_avg_ elucidated an autocorrelation range of 95 m and a relative nugget effect of 18%. The range is only slightly larger than that of the ECa mapping indicating some similarity in the spatial processes. The comparatively high nugget effect in NIR_avg_ could have resulted to some extent from measurement noise due to straylight from the sun. Since firm contact of the spectrometer shank could not always be guaranteed, sunlight may have added to the reflection causing baseline shifts in the NIR spectra. In NIR_ICA1-2_ the nugget effect was reduced or not present due to the removal of the baselines effects by differentiation of the spectra.

### Correlations between sensor readings and earthworm abundances

**Fig 4:**
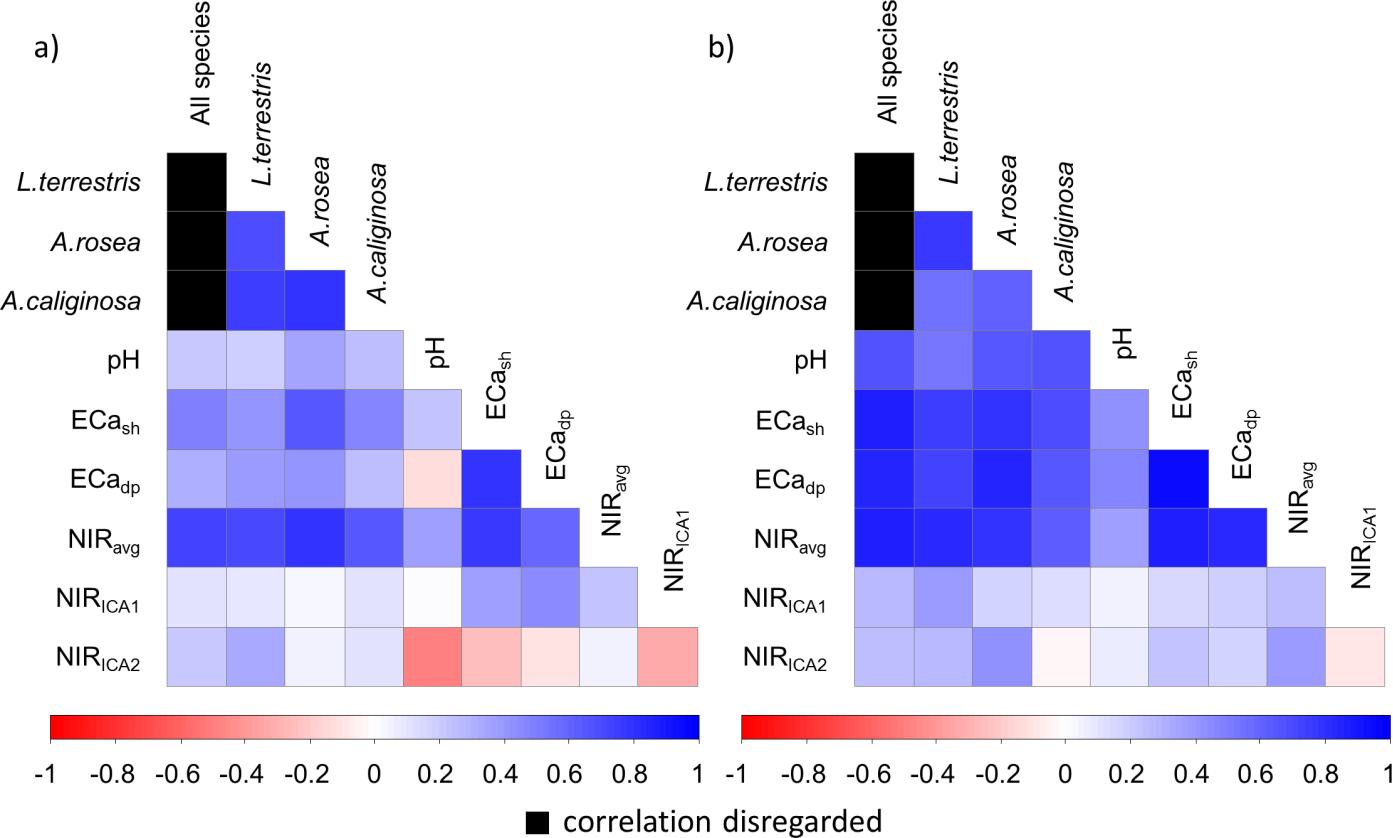
Correlation matrix (Spearman rank correlation coefficients) between sensor variables and earthworm species. (a) conventional tillage and (b) reduced tillage.

Global correlations between PSS measurements and the averaged earthworm abundances differed depending on the tillage system (Fig 4). At the reduced tillage plots, all soil sensor readings, besides the NIR_ICA1-2_, exhibited correlations (r ≥ 0.67) with the abundances of all earthworm species. Regarding the specific sensor types, pH showed lower correlation (r = 0.67) compared to ECa_sh_, ECa_dp_, and NIR_avg_. The latter ones were equally high correlated with earthworm abundances (r = 0.86). Since these sensor readings are affected by similar soil properties, cross correlation between the sensor data can be expected. Thus, partial correlation coefficients (Spearman rank) between pH, ECa_dp_, NIR_avg_, and total earthworm abundance changed correlation ranking to pH (r_part_ = 0.71) > NIR_avg_ (r_part_ = 0.69) > ECa_dp_ (r_part_ = 0.11), with ECa_dp_ not significant on p < 0.01. This ranking remained unchanged if ECa_dp_ was replaced by ECa_sh_. Even though the sensing principles are quite different, ECa and NIR_avg_ are both affected by soil texture [7,35], which in turn strongly influences earthworm activity [16]. In contrast, correlations at the conventional tillage plots were lower compared to the reduced tillage plots. Higher correlations were only reported for NIR_avg_ (r = 0.72) and ECa_sh_ (r = 0.48). Interestingly, correlation with NIR measurements seemed to be rather unaffected by the soil management.

According to the “Law of Minimum” stated by von Liebig [56] crop growth is determined by the factor that is limiting and other factors will not or very little affect crop growth. These other factors are decoupled by the limiting factor. The Law might be extended to other groups of organisms [57]. In our context, ploughing might be the limiting factor on that part of the field which is under intensive tillage. Additionally, ploughing should also be seen as a strong change of the soil environment and therefore in the habitat situation of the earthworms, since it alters among others the soil structure or soil organic matter content. Other studies [23,31] confirmed closer relations between earthworm abundances and soil properties under reduced tillage in this field.

### Global spatial modelling with generalized additive models

The GAM for the overall earthworm species (Total) described 73% of the variation as expressed by the R^2^ value (Table 3). Validation showed that the model explained 65% of the variation in the validation set with an MAE of 5.70 individuals of the total earthworm abundances. All sensors contributed significantly to the GAM. Replacing ECa_sh_ by either NIR_avg_ or ECa_dp_ in the model, resulted in similar model qualities (69% and 73% variance explained). Obviously, the EC signal and the NIR average reflectance signal were highly interchangeable for estimating the total earthworm abundances. Model quality deteriorated when fitting a GAM without NIR_ICA2_ (GVC score changed from 40.95 to 40.1), which shows that information provided by the spectral absorption peaks should not be neglected and the entire NIR spectra was useful for modelling.

**Table 3:** Generalized additive modelling (GAM) results for total earthworm abundances and the observed earthworm species with PSS as predictors. GAMs were computed with log-link function and quasi-Poisson error distribution with the observed sums at the measurement plots and averaged afterwards. The spatial coordinates of the measurement plots f(x,y) were included as a bivariate function smoothed with thin plate regression splines. Model validation was performed using leave-one-out cross validation.

Response	Predictor	p-value	sign.	Deviance explained [%]	R^2^adj.	GCV score	R^2^pred.	MAE	ME
Total counts				71.6	0.73	40.6	0.65	5.70	-0.76
	f(x,y)	> 0.1							
	EC_10_	0.000	***						
	pH	0.000	***						
	NIR_ICA2_	0.000	***						
*L*.*terrestris*				68.4	0.68	31.2	0.44	3.62	-0.99
	f(x,y)	>0.1							
	EC_10_	0.077							
	pH	0.000	***						
	NIR_ICA1_	0.003	**						
	NIR_ICA2_	0.000	***						
*A*.*rosea*				80.7	0.84	11.6	0.55	1.97	-0.96
	f(x,y)	>0.1							
	NIR	0.000	***						
	pH	0.000	***						
*A*.*caliginosa*				48.6	0.44	29.1	0.25	3.66	-0.19
	f(x,y)	0.020	*						
	pH	0.002	**						
	NIR_ICA2_	0.017	*						

Significant (sign.) at the *0.05, **0.01, or ***0.001 probability level.

The comparison of the model results for the individual earthworm species showed that the model quality declined in the order *A*. *rosea* > *L*. *terrestris* > *A*. *caliginosa* with decreasing explained variation from 84 to 68 and 44%, respectively. The differences between the earthworm species corresponded to their relation with the soil environment, which can be explained by the ecological adaptability of each species. *Aporrectodea caliginosa* is known to have the broadest ecological niche of the observed species, whereas *A*. *rosea* prefers soils with higher organic matter content in this region [58]. This elucidates the high differences in model quality between those two species. Since *A*. *caliginosa* can adapt well to variations in the soil environment, e.g., changes in soil organic matter, clay content, soil moisture, or management [35,59], proximal soil sensors are unable to reveal its habitat with reasonable spatial accuracy because the sensors detect mainly spatial variations induced by soil properties. Yet, adaptability and wide tolerance limits decoupled *A*. *caliginosa* from certain soil properties. In this case, the modelling of abundance by the inclusion of spatial coordinates proved to be advantageous, which may indicate effects of other, still unrevealed habitat factors.

The reverse was true for *A*. *rosea*. It is well known that *A*. *rosea* is a very demanding species with respect to soil organic matter supply [4,36]. If ECa_sh_ or ECa_dp_ was used as the predictor variable in the GAM instead of NIR_avg_, the model quality was inferior (GVC-score changed from NIR_avg_ to ECa_sh_ or ECa_dp_ from 11.6 to 17.0 or 19.4). This underpins the relevance of the NIR signal in assessing earthworm communities on the species level because NIR soil absorbance is strongly affected by organic substances which is present in the soil material [41].

*Lumbricus terrestris* is more adaptable to different environmental conditions and more abundant than *A*. *rosea* at the studied site. As an anecic earthworm species, *L*. *terrestris* constructs deep burrows with their own micro-environment [60]. Its burrowing activity is negatively affected by coarse particles in the soil. Thus, *L*. *terrestris* is expected to be sensitive to soil texture. Because the EC signal is related to sand and clay content it should be suitable for estimating spatial variation of *L*. *terrestris* abundances. Indeed, the inclusion of ECa_sh_ improved the model quality compared to using NIR_avg_ as the sole predictor. The lower significance of ECa_sh_ of the GAM model shown in Table 3 is due to the inclusion of the other predictor variables (model VIF = 3.15). It was expected that pH should contribute only little to the GAM model due to the eco-physiology of *L*. *terrestris*. This species can modify the redox potential in its immediate environment and is therefore less sensitive to soil pH [60,61]. However, soil pH may not be disregarded in the model as it affects bioavailability of ions, e.g., toxic elements, plant nutrients, and soil organic matter decomposition.

Because the habitat selection of earthworms is affected by many different soil properties it is most reasonable that specific sensor combinations as predictors produced the best results for predicting specific earthworm species.

**Fig 5:**
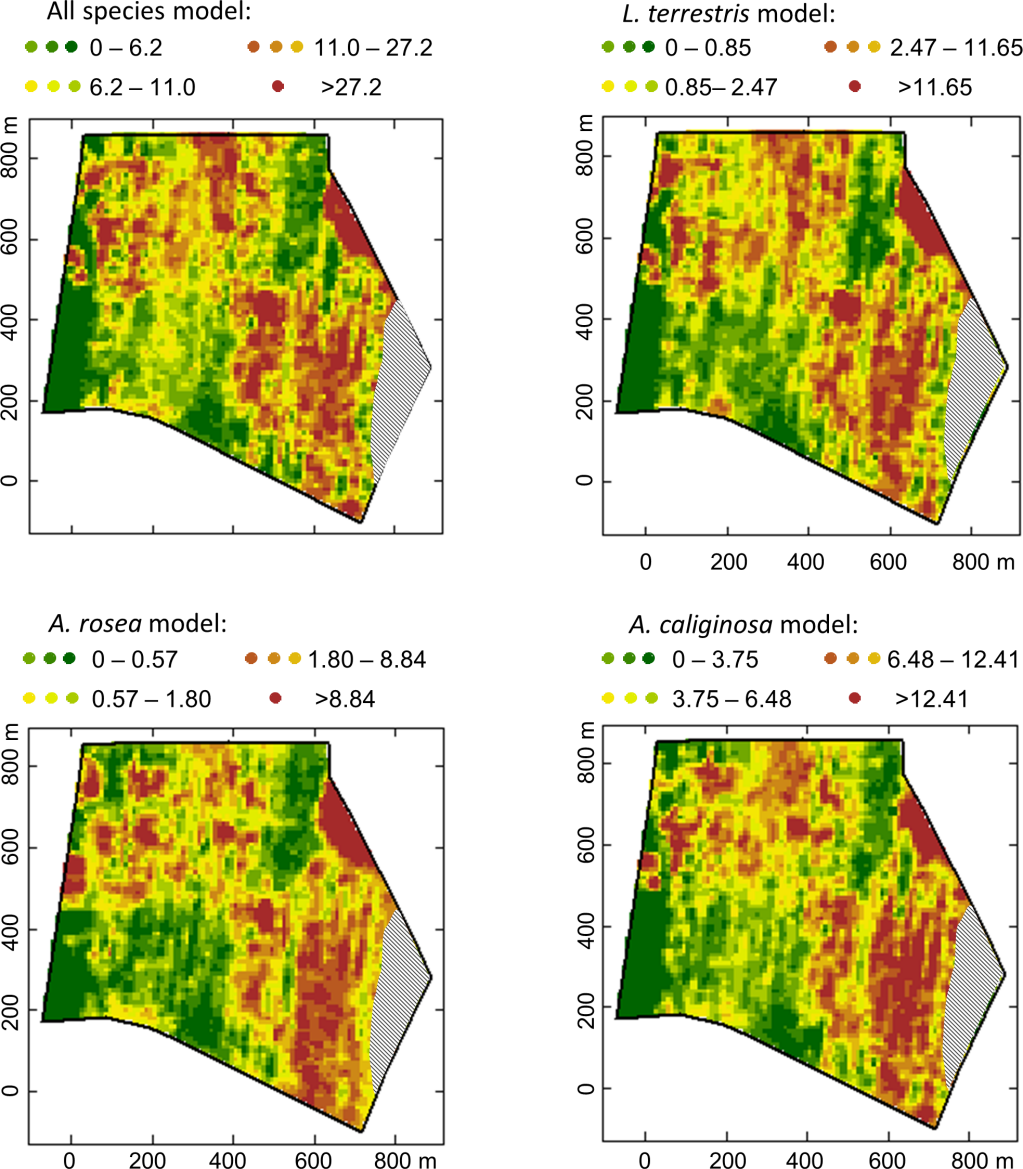
Earthworm prediction maps. Average earthworm abundances predicted by the generalized additive models as given in Table 3. Predictions were made on a 5x5 m² grid spanning the entire field. Quantile classification (10 levels).

Prediction maps of the earthworm abundances show a similar overall spatial pattern of the earthworm abundances with some typal-specific differences (Fig 5). *Lumbricus terrestris* and *A*. *rosea* are estimated to be less abundant than *A*. *caliginosa* on the sandy plateau in the south eastern part of the field. Areas with very sandy soils are expected to be completely avoided by all species. At the south western part we encountered generally more humus-enriched soils. In these soils, *A*. *rosea* should constitute its largest propagation. In contrast, *L*. *terrestris* is estimated to be more frequent at the north where the sand content is relatively low and the clay content high.

### Local spatial modelling with state-space analysis

**Fig 6:**
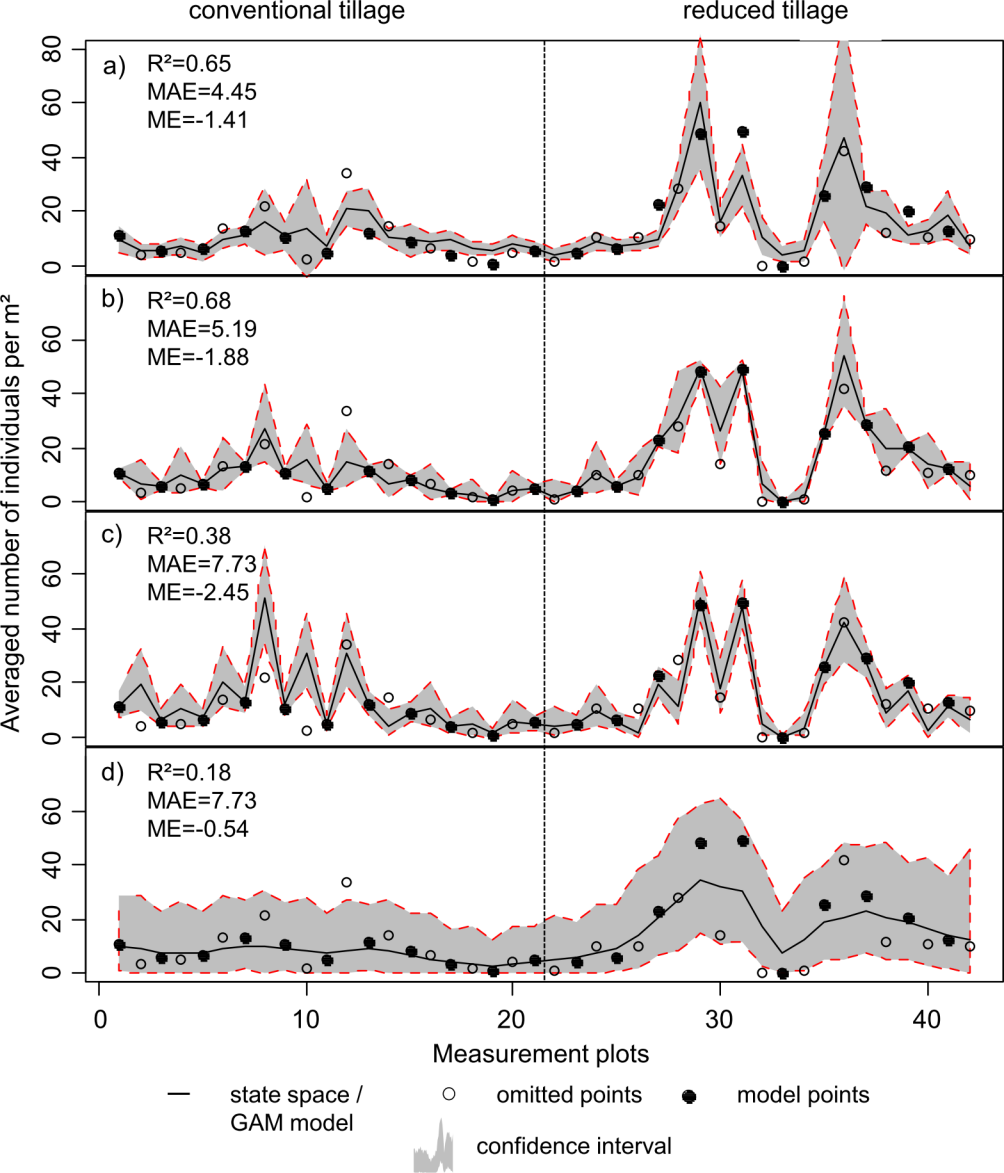
Local modelling results. (a) Estimation of average total earthworm abundances based on the GAM as given in Table 3, (b) state-space modelling (SSM) with the PSS variables as covariates as given in Table 3, (c) SSM with ECa_sh_ and ECa_dp_ and (d) SSM without co-variates. Confidence intervals were computed from the standard errors of prediction on a 95% significance level.

In Fig 6, the state-space model and the GAM model calculated for total earthworm abundances as response are shown with a) Ma: the global GAM model of the total earthworm abundances from the previous section, b) Mb: State-space model variables like in Ma, c) Mc: only ECa_sh_ and ECa_dp_ as predictors in comparison to Joschko, et al. (2010) [31] and d) Md: the earthworm abundances of the neighbouring plots as the only predictors.

The accuracy of local state-space prediction of earthworm abundances was comparatively low in Md (r^2^ = 0.18). This indicated that the spatial autocorrelation between the measurement plots was too low to estimate the abundance at the adjacent locations adequately. Earthworms exhibited a high spatial heterogeneity in this field. To estimate their spatial distribution for the entire field using only the spatial autocorrelation would necessitate a higher sampling density. The inclusion of EC as predictor did improve model accuracy to some extent (R^2^ = 0.38). Using the sensor data combination selected by the GAM model in the state space model (Mb), yielded acceptable model fit (R^2^ = 0.68). In comparison, the global GAM model (Ma) achieved similar accuracy as in the Mb. The predictive power of the latter model is comparable with the state-space model based on local data of organic carbon (0–15 cm) and fine particles content, which yielded an r^2^ of 0.70 over the same measurement plots [23].

The tillage regime has a strong effect on the performance of prediction models based on sensor data. Under conventional tillage the models were less accurate than under reduced tillage. Specifically, Mc had a quite “spiky” appearance with large model residuals. In particular, at measurement plots 8 and 10, predictions based on EC sensor data were poor. Similar differences in estimation accuracy of ECa data between conventional and reduced tillage were reported by Joschko et al. (2010) [31]. In contrary, the multi-sensor model (Mb) was more robust and its predictions were more accurate compared to Mc. However, Mb in the ploughed portion of the field is still less accurate compared to the model’s predictions in the reduced tillage plots, despite the strong fluctuations of abundances among the measurement plots in reduced tillage. At some locations, the sole inclusion of EC sensor readings (Mc) yielded even better results than with the prediction by data from the multiple sensors (Mb).

The larger estimation errors in the conventional tillage area further confirms the decoupling of earthworm occurrence and natural soil properties under this tillage regime. State-space models do perform better with data from the reduced tillage area, since the influences of natural factors are not cancelled out completely by human activity (ploughing) [23]. As proposed by Fox et al. (2004) [62], it can be assumed that soil differences such as soil texture or soil organic matter are modulated by management practices and together they will impact the basic abundance pattern of earthworms in agricultural soils. Under tillage systems, which decouple the earthworm-soil relationship, PSS cannot model earthworm abundances adequately, whereas under tillage systems, which preserve the earthworm-soil relationship, PSS seems well suited to predict earthworm abundances.

## Assumptions, Limitation and Weaknesses of This Study

The use of soil sensors in a field under regular management and the combination of instantaneous soil sensor measurements with an already established long-term earthworm field experiment as it was done in this study may give rise to some problems.

Due to management schedule of the Komturei Lietzen, we had only a short timeframe were the sensor measurements could be conducted within late summer where we encountered hot air temperature. Although soil sensor measurements were conducted accurately and the weather conditions and the dry soil condition did not alter the sensor measurements in a wrong way, the spectrophotometer had to be cooled down for some time, which prolonged the measurement campaign over several days. Under lower temperature conditions, the campaign would have been certainly finished in one day. Moreover, the use of soil sensors is not without any issues especially when integrated in a sensor fusion. Thus, severe sensor failures (pH) happened during the campaign but only in a very small part of the field at the western boundary. These measurements were taken out. Measurement plots were at least 270 m away from these failures and we do not assume that they were influenced by those missing values. Of course, sensor measurements could be only done at those locations where tractor passage was applicable. Due to water ponding in the middle of the field, a small measurement free area exists. However, sensor measurements were conducted as far as possible to the edge of the water pond, which can be seen in Fig 3, because ECa values suddenly rise due to the effect of higher clay content and soil moisture. But soil data within the area of water ponding could not be attained and is therefore missing in the dataset. We interpolated those values. The water ponding in this area is constantly observed over the years within this field and it has to be said that PSS is generally bound to some practical limitations, which might result in some missing values in the final map representation.

Furthermore, PSS will not deliver highly accurate soil measurements as given by manual soil sampling and lab analysis. Its main advantage is its high spatial resolution which can be reached with an automatic measurement principle but can be quite different depending on the type of sensor used. The choice of soil sensor we used in this study is representative over a wide range of different PSS principles including optical, electrochemical, and geo-electrical approaches. However, all these sensors come with its own sampling support and sampling frequency depending on its measurement principle. This is implicit to the sensors used and cannot be influenced. However, geostatistical techniques are able to counteract differences in sample support and sampling frequency to a certain extent by using Kriging interpolation [38].

We selected the Lietzen site for this study because we had the advantage of a large monitoring study of earthworms over 11 years. Although the soil heterogeneity observed in this field is representative for many fields in this region, it is clear that the study is nevertheless somewhat limited to sandy and loamy soils in a ground moraine landscape. Earthworms show generally a very low abundance within sandy soils. The use of earthworm observations of only one year ends up in data sets with lots of zero counts at the measurement plots. By using the monitoring data set of the Lietzen site, we were able to get a better estimate of the earthworm occurrence at each measurement plot by averaging the 11 year observations. We generally accept that the average value recognizes the potential of earthworm abundance over a long period of time at each measurement plot. For the validness of this study, we assumed that this potential of the earthworm-soil relationship still applies in a same way at the time of the PSS measurement as at the time of the earthworm monitoring. We further assume that there is a relationship between the soil properties during 1997 and 2007 and the sensor data. To support this assumption, we correlated data of soil organic carbon (SOC) and clay content (CC) measured around the time period of the long-term earthworm field experiment with the PSS data (S2 File, S1 Table). We determined highly significant correlations for SOC with NIR and pH sensor data (p < 0.001) whereas less significant correlations for ECa data (p < 0.01). For CC the correlations were higher than for SOC (p < 0.001) in the declining ranking ECa > NIR > pH (S1 Table).

## Conclusions

Earthworm observations averaged over 11 years show a high spatial variability of abundances. To capture this variation within the entire field by manual sampling of earthworms would imply a tremendous sampling effort. If the underlying process of the spatial distribution of earthworms is driven by natural soil properties, proximal soil sensor data show strong correlations with earthworm abundances even at the species level. However, correlations were weaker when tillage disturbed the soil–earthworm relationship. It was further shown that spatial data provided by PSS is able to reasonably estimate earthworm abundances with GAM as well as state-space models. Therefore, introducing PSS data into field scale SDM would increase model quality with less sampling effort.

At the individual species level, the choice of the appropriate sensor constellation is important due to the different habitat preferences of the respective earthworm species. Since the abundance of *L*. *terrestris* is sensitive to changes of sand content, the inclusion of the EC sensor data produces better estimates. In contrast, ECa data is not necessary for *A*. *rosea*, where NIR and pH are more meaningful predictors. This is caused by its strong relationship with soil organic matter. Therefore, the complexity of habitat selection of individual earthworm species advocates a multi-sensor approach to estimate the spatial distribution of earthworm abundances. However, for an overall assessment or approximation, the use of the NIR sensor is recommended.

Nonetheless, PSS data is only a proxy describing the spatial patterns of important soil properties influencing the earthworm habitat such as soil texture or organic matter. Likewise, measurements of soil texture and organic matter are only proxies describing broadly aggregate stability, above- and below-ground plant decomposition, macropore volume, or nutrient availability, which more directly influence the earthworm habitat. However, direct sensor measurements of earthworms are difficult to attain and should be the focus of future research in PSS. For now, PSS has potential as a spatial predictor in field-scale SDM for earthworms. Moreover, PSS can be used for direct the sampling of earthworms within fields or for designing a more sophisticated field experiment implementing the PSS data as prior spatial information about the within-field variability of the soils.

## Supporting Information

S1 File PSS data and earthworm abundances. This file contains the PSS data as interpolated onto the measurement plots, the total sums of the earthworm abundances observed between 1997 and 2007, and the coordinates of the plot locations.
(CSV)

S2 File Soil organic carbon (SOC) and clay content (CC). This file contains averaged data of the SOC and CC data. Samples for SOC were taken in 1996, 2004, 2005, 2006, and 2008 at the measurement plots and analyzed for total carbon after dry combustion at 1250°C using a CNS-2000 analyzer (LECO, Ltd., Mönchengladbach, Germany). Carbonate carbon was determined after treatment with phosphoric acid by electric conductivity measurement of carbon dioxide. SOC was calculated as the difference of total C and carbonate carbon. Samples for CC were taken in 2004. CC was determined by wet-sieving and sedimentation with Köhn-Pipette method after organic C destruction with H_2_O_2_ and chemical dispersion using Na_4_P_2_O_7_.
(CSV)

S1 Table Correlation (Pearson) between PSS data and soil parameters. Soil parameters were determined during the long-term earthworm field experiment as described under S2 File.
(DOCX)
