# Supplementary material for: Proximal Soil Sensing – A Contribution for Species Habitat Distribution Modelling of Earthworms in Agricultural Soils?
Source: PLoS One. 2016 Jun 29;11(6):e0158271. doi: 10.1371/journal.pone.0158271 (PMC4927140; doi:10.1371/journal.pone.0158271)
Supplement: S1 Table — Soil parameters were determined during the long-term earthworm field experiment as described under S2 File. (DOCX) [file pone.0158271.s003.docx]

S1 Table. Correlation (Pearson) between PSS variables and soil parameters. Soil parameters were determined during the long-term earthworm trial.

| Sensor | C_org_ | Clay content |
| --- | --- | --- |
| pH | 0.51^***^ | 0.58^***^ |
| ECa_sh_ | 0.40^**^ | 0.84^***^ |
| ECa_dp_ | 0.44^**^ | 0.73^***^ |
| NIR | 0.57^***^ | 0.77^***^ |
| NIR_ICA1_ | 0.38^*^ | 0.18 |
| NIR_ICA2_ | 0.38^*^ | 0.02 |

Significant at the *0.05, **0.01, or ***0.001 probability level.
